# Cardiac Rehabilitation practitioners’ views on patients’ psychological needs: a qualitative study

**DOI:** 10.3389/fpsyt.2024.1434779

**Published:** 2024-10-03

**Authors:** Laura Wray, Lora Capobianco, Adrian Wells

**Affiliations:** ^1^ Division of Psychology and Mental Health, School of Health Sciences, University of Manchester, Manchester, United Kingdom; ^2^ Research and Innovation, Greater Manchester Mental Health National Health Service (NHS) Foundation Trust, Manchester, United Kingdom

**Keywords:** Cardiac Rehabilitation, mental health, qualitative, anxiety, psychological needs

## Abstract

**Background:**

Psychological difficulties are prevalent in patients undergoing Cardiac Rehabilitation (CR). Recent guidelines recommend that practitioners inquire and address patients’ psychological concerns during CR. Therefore, Study One aimed to explore practitioners’ understanding of patients’ psychological needs, their confidence in supporting those needs, and their views on whether current CR meets patients’ needs. Study Two aimed to validate Study Ones’ findings among a wider sample of CR practitioners.

**Methods:**

This study consisted of two interrelated qualitative interviews. Study One utilised qualitative interview data from the PATHWAY trial (REC Reference:15/NW/0163), while Study Two utilised new interview data collected as part of the PATHWAY Beacons study (REC Reference: 22/HRA/2220). In Study One semi-structured interviews with six CR practitioners were analysed using thematic analysis. In Study Two, 11 CR practitioners across England were interviewed using member-checking principles. Transcripts were coded systematically using the codes developed in Study One and, through constant comparative analysis.

**Results:**

Four main themes were identified: staff’s awareness of mental health problems, CR patients’ needs, staff’s self-efficacy to support patients’ psychological needs, and current psychological provision in CR. The main themes and 11 subthemes were transferable to a wider range of CR practitioners, thereby indicating the trustworthiness of the findings.

**Conclusion:**

Practitioners described that patients experience a range of psychological concerns, including adjustment difficulties, anxiety, and cardiac and noncardiac worries. Most practitioners normalise patient concerns and offer relaxation techniques. However, practitioners have noted that patients often have complex psychological needs, but practitioners’ confidence in discussing and supporting psychological concerns varies. Practitioners expressed the need for training to support patients’ psychological needs.

## Introduction

Cardiac Rehabilitation (CR) is a structured rehabilitation programme, designed to reduce the physiological and psychological effects of cardiac illness in patients following heart attack, heart surgery, or revascularisation, as well as in those with stable heart failure (1). Only 52% of eligible patients attend CR across England, which equates to 62,822 patients (2). However, the national strategic plan aims for uptake to reach 85% by 2028 (3), as evidence, including systematic reviews and meta-analyses, indicates that CR is beneficial in improving patients’ quality of life (4) and reducing morbidity and mortality (5–7).

While one of the main priorities of CR is improving patients’ physical recovery, CR is widely seen as a vehicle for delivering psychological interventions to cardiac patients (8–11). Anxiety and depression are common in CR; approximately 28% of patients experience clinically significant symptoms of anxiety and 18% experience clinically significant symptoms of depression (2, 12). For some patients, their psychological difficulties are transient and settle around three months post-cardiac event (13), whereas for others, their difficulties become more longstanding, with varying levels of complexity.

Anxiety and depression in cardiac patients are associated with increased mortality, decreased quality of life and reduced CR attendance and adherence (14–18). Key policies (19–21) and international guidelines (10), have outlined the need for integrated physical and psychological care. While policies support the integration of psychological care in healthcare, guidance on psychological provision in CR is not well-defined (22). The main psychological provision consists of relaxation and stress management talks, typically provided by CR practitioners who have limited psychological training and are often constricted by time due to the broad curriculum of CR (19). This results in many patients failing to receive satisfactory psychological care during CR.

The British Association for Cardiovascular Prevention and Rehabilitation (BACPR) standard (8) states that psychosocial health should be explored during the initial CR assessment to ensure that patients’ individual needs are supported. However, only over half of the patients were asked about their mental health (23). As there are a limited number of psychologists commissioned to work in CR (19), initial psychosocial assessments are usually completed by CR practitioners. However, research indicates that practitioners have difficulty identifying depression and panic in cardiac patients (24–26) as somatic symptoms can mirror medical conditions and medication side effects. This suggests that practitioners lack sufficient skills to consistently assess patients’ psychological experiences and identify those who may benefit from an onward mental health referral. Furthermore, CR does not routinely offer psychological intervention to target anxiety or depression, indicating that patients’ psychological needs are not met.

To our knowledge, only two studies have interviewed CR patients with clinical distress to explore their psychological needs (27, 28). Turner et al. (27) explored the views of CR nurses, and patients who had screened positively for depression, about providing, and receiving psychological care in CR. They noted that patients described significant changes in their emotional well-being following the cardiac event, such as experiencing panic attacks, lower self-confidence, and a sense of loss due to changes in what they could do before compared to after the event. Similarly, McPhillips et al. (28) evaluated how patients with depression and/or anxiety described their psychological needs and their views on how CR addressed their psychological needs. They found that patients described feeling low in mood along with varying concerns, including worrying about having another cardiac event and the impact that the cardiac event had on their lives. Patients described their worry as constant, and felt that worrying was uncontrollable and harmful. Some patients described seeking reassurance from other patients and practitioners to ensure that their experiences were normal, while others felt uncomfortable discussing how they felt with the practitioners. Most notably, in both studies, patients felt that psychological support from practitioners was limited.

To date, little is known about CR practitioners’ understanding of patients’ psychological needs. It is imperative that practitioners understand patients’ psychological needs to provide holistic care, align with current policies (20) and standards (8), and work towards psychologically informed care in CR. Qualitative research can be utilised to explore healthcare needs (29), but it is important to generate trustworthy findings. To support this, previous research has utilised member checking, a process whereby qualitative findings are presented to participants to check if the findings resonate with their experiences (30, 31). Member checking can be used to explore whether qualitative findings are credible and transferable across populations (32, 33). As such, the current study utilised two nested qualitative interview studies incorporating an initial interview followed by a second member-checking interview, which are reported separately below. In Study One, our aims were to (1): establish CR practitioners’ understanding of CR patients’ psychological needs (2); establish practitioners’ confidence in supporting patients’ psychological needs; and (3) to establish practitioners’ views on whether current CR meets the psychological needs of its patients. In Study Two, our aim was to assess the credibility and transferability of the themes and subthemes generated in Study One among a wider sample of practitioners, working across six CR services in England. We also sought to establish additional views and experience. By assessing credibility and transferability, we aimed to validate the themes of Study One.

## Study one

### Design

The current qualitative study was nested within the PATHWAY trial (34, 35). Data were collected as part of the PATHWAY trial, where CR practitioners were trained to deliver group-metacognitive therapy (MCT). A qualitative research design using semi-structured interviews, supported by an interview guide, was used. Practitioners were interviewed at three time points: prior to training in group-MCT, during, and after training. Only data from the first time point were used to ensure that the data were not influenced by training. This study is underpinned by an essentialist framework to explore practitioners’ views in an understudied area, whereby meaning is based on a relatively straightforward relationship between experience and language (36).

### Participants

Purposive sampling was used to recruit participants as only practitioners enrolled in the PATHWAY trial were eligible for inclusion. Ten practitioners from five CR services in Northwest England agreed to be trained in group-MCT and consented to be interviewed at the three time points. However, as the two CR services joined the trial later, there was insufficient time to schedule an interview prior to training in group-MCT. Six practitioners were interviewed before training. The participants included nurses (n = 3), one physiotherapist, one occupational therapist, and one exercise facilitator. All practitioners were female, with an average age of 46.2 (range: 28–58 years), and the average length of time working in CR was 8.6 years (range: six months to 24 years). None of the participants withdrew from this study. The practitioners’ characteristics are listed in **Table 1**.

**Table 1:** Study One participant characteristics.

Participant Number	NHS Site	Profession	Sex	Age	Length of time working in CR (years)	Any previous mental health training (not MCT)	Prior training in group-MCT
1	B	Physiotherapist	F	28	4	N	N
2	C	Nurse	F	47	15	N	N
3	A	Occupational Therapist	F	39	0.5	Y	N
4	C	Exercise Facilitator	F	51	3	N	N
5	A	Nurse	F	58	5	N	N
6	B	Nurse	F	54	24	N	N

### Data collection

All interviews were structured using an interview guide, which contained a mixture of open-ended and more directive questions, and were conducted face-to-face in a confidential space by a female qualitative researcher between January and February 2015. The CR practitioners were asked about their perceptions of patients’ psychological needs, their views on supporting psychological needs, and how CR supports psychological needs. On average, interviews lasted 46 min, with interviews ranging from 37 min to 55 min, producing a total of 275.53 min of data. The interviews were audio-recorded and transcribed verbatim, with identifiable information removed by a third-party organisation.

As pre-collected interview data were utilised, an audit trail and a quality check of the dataset were completed. Following this, the researchers made a collective decision that sufficient data had been collected to generate meaningful analysis, as data adequacy goes beyond the sample size (37).

### Ethical considerations

The data for this study were collected as part of the PATHWAY trial. Ethical approval was granted by the UK Health Research Authority, Northwest Centre of Research Ethics Committee (REC reference: 15/NW/0163). All participants were provided with an information sheet and a consent form prior to participation, and all provided written consent.

### Data analysis

LW conducted a secondary analysis of the interview data. The data were analysed using thematic analysis (36, 38). LW familiarised herself with the data by listening to audio recordings and reading transcriptions numerous times. The initial codes were generated inductively and were not driven by a predetermined framework. As the analysis was not linear, an iterative process was used (39), whereby LW coded the data within each transcript relevant to the study aims. LW, LC, and AW discussed the codes generated, and discrepancies were resolved. Candidate themes were discussed by all authors, and semantic themes were identified on agreement.

### Trustworthiness and reflexivity statement

The primary researcher, LW, was a trainee clinical psychologist with clinical experience working with multidisciplinary teams in health psychology services. AW is the originator of the MCT and LC is a registered MCTI therapist. The authors did not have any personal or professional relationship with the participants. The research team comprised one male (AW) and two females (LW and LC), and acknowledged that researchers are intertwined with personal, clinical, and academic pursuits. To enhance trustworthiness, LW kept a reflective diary throughout the research process to develop awareness of personal assumptions and outline the methodological and analysis decisions made by the research team. The COREQ checklist (40) was adhered to (**Appendix 1**).

## Findings

**Figure 1:**
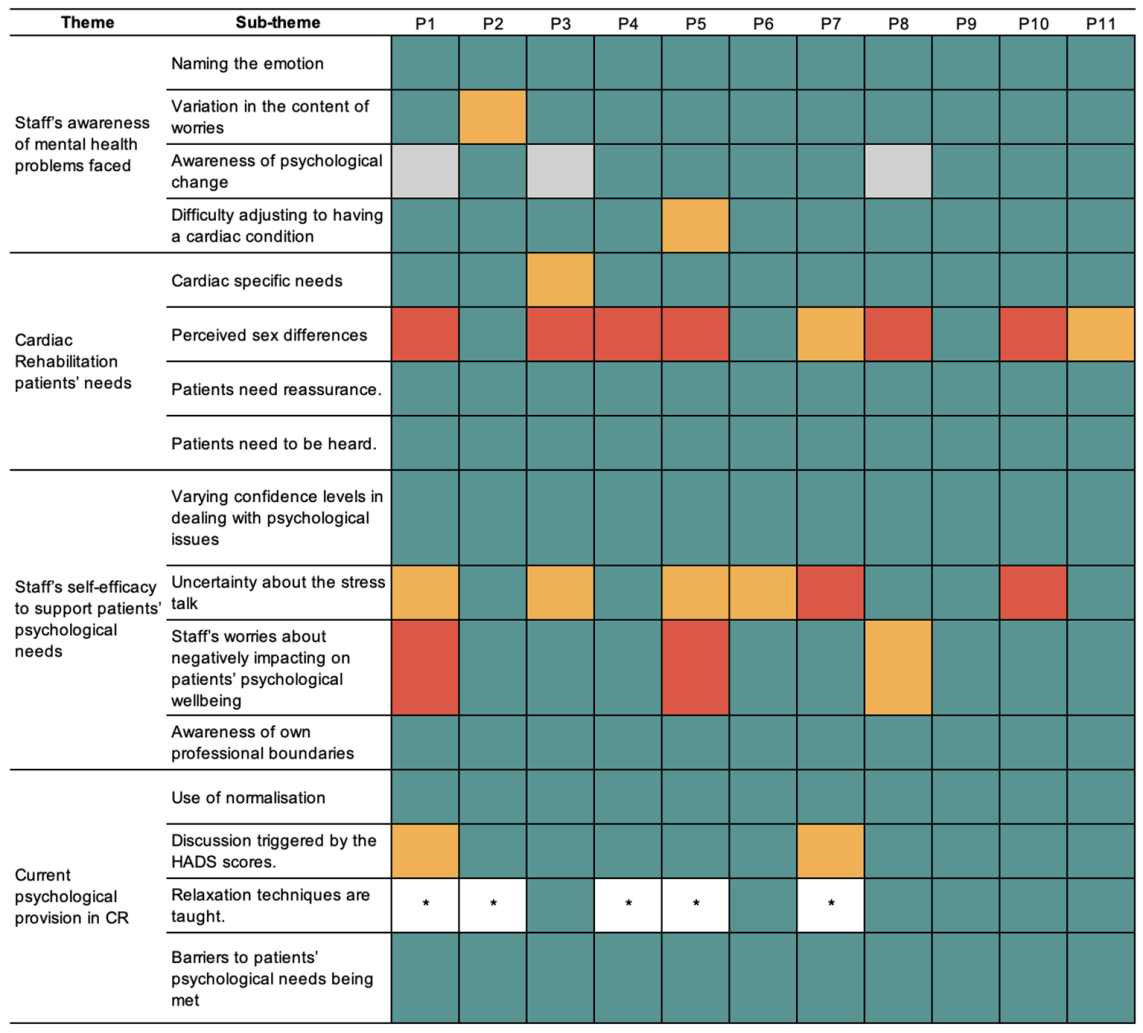
Thematic map. Dotted line, relationship between themes; solid line, link to sub theme; circle, theme; square, sub theme.

Four main themes were generated (1): staff’s awareness of mental health problems (2), CR patients’ needs (3), staff’s self-efficacy to support patients’ psychological needs, and (4) current psychological provision in CR. Each theme encompasses four subthemes as outlined in **Table 2** and **Figure 1**. Interview excerpts were also included to support these findings. Illustrative quotes have been provided.

**Table 2:** List of themes and subthemes.

Main Themes	Subthemes
Staff’s awareness of mental health problems faced	• Naming the emotion • Variation in the content of worries • Awareness of psychological change • Difficulty adjusting to having a cardiac condition
Cardiac rehabilitation patients’ needs	• Cardiac specific needs • Patients need to be heard. • Patients need reassurance. • Perceived sex differences*
Staff’s self-efficacy to support patients’ psychological needs	• Varying confidence levels in dealing with psychological issues. • Uncertainty about the stress talk • Staff’s worries about negatively impacting on patients’ psychological wellbeing. • Awareness of own professional boundaries.
Current psychological provision in CR	• Use of normalisation • Discussions triggered by HADS scores. • Relaxation techniques are taught. • Barriers to patients’ psychological needs being met.

*following Study Two this theme evolved and was re-named ‘perceived individual differences.’

### Theme 1: Staff’s awareness of mental health problems faced

The first theme concerns practitioners’ awareness and understanding of the psychological changes and difficulties patients typically experience. The theme encompasses four subthemes: practitioners’ ability to name the emotions experienced by patients, practitioners’ reflection on the adjustment to having a cardiac condition, and practitioners’ ability to note that patients experience a psychological change and a variation in the content of their worries.

#### Naming the emotion

Practitioners were able to highlight the emotions experienced by patients, noting that they often experienced stress, anxiety, fear, and low mood. Practitioners perceived patients experience a *‘fear factor’* that *‘everything they do could trigger off another [cardiac] event’* (6B—Nurse) but highlighted that some patients find it difficult to name the emotion they are personally experiencing. From clinical experience, practitioners perceived *‘anxiety and low mood would tend to be the more common ones [mental health difficulties]’* (6B—Nurse) patients’ experience.

#### Difficulty adjusting to having a cardiac condition

Practitioners reflected on how a cardiac event can be a *‘massive shock’* for patients, especially if they perceive themselves to be healthy. Practitioners noted that patients need to adjust from *‘thinking they’re fit and healthy’* (5A—Nurse) to being diagnosed with a long-term health condition. Practitioners noted that there are individual differences in the way patients adjust to a cardiac event. One practitioner indicated that some patients do not appear phased and adjust well;

“I mean some people it’s just like water off a duck’s back, they have a positive attitude towards it [having a heart attack]” (2C—Nurse).

#### Awareness of psychological change

Practitioners noted patients often experience negative psychological changes following a cardiac event, such as increased worry, and exacerbation of pre-existing anxiety and depression;

“Things that weren’t worrying them before might be worrying them now and the things that might have worried them a little, it might be worrying them a lot more now.” (1B—Physiotherapist).

Practitioners report noting how patients present with an increased awareness of their bodily sensations, especially following exercise or when they feel anxious, which can ‘*hinder their physical ability.’* (3A—OT), as they found it hard to engage in CR due to fear of reoccurrence.

“[They are] worried about having pains in their chest … or breathlessness … they are often hypersensitive to the area … so it is often anxieties around pains, twinges … they wouldn’t have even noticed twinges and slight breathlessness before, but now they are thinking … noticing it.” (1B—Physiotherapist)

Practitioners noted that there is sense that some patients often experience heightened psychological difficulties initially,

“we would look at the psychological reaction and normalise that for patients … and explore all the various feelings that … might be very strong in the early days (6B—Nurse).

Practitioners reflected that, for some patients, their psychological difficulties subsided throughout CR.

“It’s quite gratifying for them to tell us that they are moving on … from the event that has happened, be it heart attack or surgery … feeling better physically, psychologically.” (5A—Nurse).

#### Variation in the content of worries

Practitioners described how patients experienced a range of cardiac and non-cardiac-related concerns, such as worry about health, finances, and daily activities. Patients expressed concern about ‘*what the future holds for them’* (3A—OT). Practitioners noted that patients worry about re-occurrence, especially when they misinterpret their bodily sensations;

“Many patients will experience slight chest pains and it will be that they think they are having another heart attack” (1B—Physiotherapist).

Practitioners observed that some patients stopped performing daily activities because of misconceptions. One practitioner noted, some patients withdraw as they believe

“I can’t do anything anymore, I will have to give up work, I can’t do this, I can’t do that” (4C—Exercise Facilitator).

Interestingly, practitioners recognised that patients’ worries go beyond cardiac-related concerns:

“It may not just be their recent cardiac event … I see people that may have recently been bereaved so there is a bereavement reaction going on or I see people with other significant co-morbidities, we see people with cancer who have now had a heart attack, financial problems can be a big one … that causes a lot of stress so … additional causes over and above their illness.” (6B—Nurse)

### Theme 2: CR patients’ needs

The second main theme centres on practitioners’ understanding of the range of psychological needs that patients experience. The theme encompasses four subthemes: cardiac-specific needs, perceived sex differences, patients need reassurance, and patients need to be heard. Underpinning the subthemes, practitioners reflected on the importance of developing a positive rapport with patients, so that patients feel able to talk about their mental health concerns.

#### Cardiac specific needs

Practitioners have noted that patients’ needs can vary between cardiac diagnoses or procedures. For example, an occupational therapist commented that patients with heart failure *‘may look physically well’* (3A-OT); however, these patients need to be supported to improve their confidence and express themselves, as others’ perceptions can make social interactions challenging and be a source of anxiety.

Practitioners believe that patients who have experienced a heart attack or require urgent procedures often receive limited advice on how to manage their condition. As such, practitioners recognise that these patients require education and correction of misconceptions to support their physical and psychological recovery:

… “So often they’re told don’t do anything until you go to cardiac rehab and people will literally not make a cup of tea … it is just making them de-conditioned for longer … and get more worries … when they come to rehab, we can educate them and give them advice and do some exercises with them and show them what they are able to do they can feel a lot more empowered after” (1B—Physiotherapist)

#### Perceived sex differences

Several practitioners have described the differences in how men and women discuss their psychological concerns. More men, typically, attend CR and some practitioners noted that men can appear to be more hesitant to discuss potential psychological difficulties compared to women;

“Men will like, try and brush it off … whereas women will be like well yes I can tell you why that is [a high HADS score]” (1B—Physiotherapist).

However, practitioners believe that developing strong rapport with patients helps facilitate conversations on mental health. One practitioner reflected on the power of talking to a male patient about their mental health, despite not initially reporting any psychological difficulties;

“[I] had a chat with him after [the group] … he’s felt very low for like thirty, forty years and he’s never spoken to anyone about it [his mental health] before” (3A—Occupational Therapist).

#### Patients need reassurance

Most practitioners indicated that patients seek reassurance and hold the belief it *‘is what a lot of people do need”* (2C—Nurse). Practitioners understand that patients tend to seek reassurance to alleviate their worries related to their physical symptoms, medication, and fear of reoccurrence. Practitioners also use reassurance to correct misconceptions held by patients or their families, to help patients improve their functioning.

“His father told him he would now have to change his job … and it is explaining to him that actually, he can get back to his way of life. We are just looking at now checking out his heart and reassuring him, come to cardiac rehab, test your heart, they do your blood pressure and your heart rate and there is no reason why you can’t get back to the occupation you were doing before.’ (5A-Nurse).

A few practitioners reflected that the initial positive effects of reassurance, such as easing patient worries by providing education on medications or symptoms, are often transient and can result in patients relying on reassurance;

“They’d be fine with us, then they’d go, and they might ring us up, say … I was short of breath, what do you think it is, palpitations … so again you try and reassure them” (2C—Nurse).

#### Patients need to be heard

All practitioners indicated that patients valued the opportunity to talk about their experiences and to be listened to. Practitioners believe that supporting and listening to patients is a core part of their role;

“Listening … is a big factor in all of it, my clinical knowledge and experience … simple things like compassion, empathy … I think to have the time where someone feels they are being listened to is really important” (2C—Nurse).

When patients feel heard, practitioners noted a positive effect on psychological recovery;

“You can see significant changes just after 2 to 3 weeks because you’ve allowed them to open up and you’ve listened to them” (6B—Nurse).

Peer support was found to be a valuable way for patients to feel understood and not alone in their experiences. One practitioner often attempts to facilitate support between patients;

“I might buddy people up, you know share with the new patient that a previous patient felt like they did, but where they are now, and you know sit them together.” (6B—Nurse).

### Theme 3: Staffs’ self-efficacy to support patients’ psychological needs

The third theme centres on practitioners’ perceptions of their ability to support psychological needs. The theme encompasses four subthemes: varying confidence levels in dealing with psychological issues, staff’s worries about negatively impacting patients’ psychological wellbeing, uncertainty about the stress talk and awareness of their own professional boundaries.

#### Varying confidence level in dealing with psychological issues

Some practitioners felt out of depth when faced with patients’ psychological issues, whereas other practitioners felt *‘suitability equipped’* (1B—Physiotherapist). Practitioners described how lack of formal mental health training, impacts their confidence and skill set;

“I have got no training on the psychological problems of addressing what has happened to them, all I can do is try and offer support.” Lack of training and low confidence can lead practitioners to *‘panic a little’* if patients start to discuss their mental health (4C—Exercise Facilitator),

despite believing supporting patients’ psychological needs as a core part of their role. One nurse reported feeling helpless and like a failure due to limited confidence, and skills, to support patients’ psychological needs;

“you feel like you are failing them in some respects so you feel like there is nothing you can do.” (2C—Nurse).

#### Staff’s worries about negatively impacting on patients’ psychological wellbeing

Interestingly, a few practitioners reported worries about asking patients about the psychological difficulties they may experience. This reticence appeared to stem from practitioners wondering if asking patients about their worries increases the time they are engaging and thinking about their worries, and subsequently may leave patients feeling worse.

“I don’t know if I’m helping them or making them think more or longer … I might not be giving them the right information. Giving them too much information might make them think about it” (4C—Exercise Facilitator)

Practitioners were also concerned about information giving, as it may be counterintuitive as it could create additional worry if patients generalise the advice.

“All you can do is show them because this artery is blocked here … so I don’t want them to be thinking too deeply that there are other arteries in other places obviously so are they going to think like that now that their other arteries are going to be blocked” (4C—Exercise Facilitator)

#### Uncertainty about the stress seminar

In UK CR services, patients often receive educational seminars on stress management. The content of stress seminars varies across services, as there are no structured guidelines on session content. Practitioners indicated that they felt that the stress talk did not typically meet patients’ needs. Indicating a formulation-based approach to treatment is not utilised within CR;

“I am always a bit sceptical about the stress talk as to how effective it is … I sometimes feel as if it too generalised … I don’t feel that confident at the end that I have done what I needed to do.” (2C—Nurse).

Uncertainty appears to negatively impact practitioners’ motivation to deliver the session;

“Stress one we all try to shun away from in some respects” (4C—Exercise Facilitator).

The uncertainty around the relevance and effectiveness of the talk might be heightened as

‘sometimes … [we] don’t get anyone coming to the [stress] talk’ (1B—Physiotherapist).

#### Awareness of own professional boundaries

Practitioners reflect when they perceive patients’ needs to be more complex, they remain mindful of working within their competence level;

“There is a ceiling of support that I can give people and beyond that it’s looking elsewhere really.” (1B—Physiotherapist).

None of the practitioners interviewed had a psychologist embedded in their CR service, as such practitioners acted as gatekeepers and referred patients to their GP or local mental health services if they scored highly on the Hospital Anxiety and Depression Scale (HADS). Some practitioners held views on certain presentations being more likely to need an onward referral; for example, for patients who have had

‘a full-blown cardiac arrest, they can be very distressed … and that is where I think they need more specialist psychological intervention’ (6B—Nurse).

Despite this, practitioners reflected

‘a lot of them [patients] would say no [to an onward referral]’ (2C—Nurse),

as such there is a sense CR is best placed to support patients’ psychological needs.

### Theme 4: Current psychological provision in CR

The fourth theme centres on practitioners’ views on the current provision of CR and whether it meets patients’ psychological needs. The theme encompasses four subthemes: discussion triggered by HADS scores, use of normalisation, relaxation techniques, and barriers to patients’ psychological needs being met.

#### Discussion triggered by HADS scores

Within CR, practitioners feel they “always ask patients about their anxiety and depression levels; we do enquire about their emotional health” (1B—Physiotherapist).

These conversations are typically facilitated when patients score high on the HADS, which relies on them completing it honestly and returning it to practitioners. In some instances, the content of these discussions is limited to exploring the statements on the HADS; rather than utilising clinical interview skills to develop a deeper understanding of the patients’ psychological experiences. Practitioners understand that some patients are open to discussions about their mental health, whereas others *‘really shut it down’* (3A—OT). Interestingly, one practitioner expressed that

“in this kind of service [CR] people have maybe never had any kind of mental health interaction” (3A—OT),

which offers a potential explanation for why some patients may find these conversations difficult.

#### Use of normalisation

The use of normalisation appears to be embedded within routine practice in attempts to help patients understand that other patients also experience new, or heightened, emotions. For example, some practitioners highlight stress as *‘normal,’* and *‘it happens to everyone’* (1B—Physiotherapist). This generalisation can fall into practitioners telling patients there is a right and a wrong way to experience stress following a cardiac event;

“I would probably just say to them you know about stress, what is normal and what’s not normal to be feeling.” (1B—Physiotherapist).

Some practitioners appear to dismiss patients worries by telling patients who are

‘very aware of any ache pain or twinge in their chest after a heart attack’ that ‘*it is nothing to worry about”* (2B—Nurse).

Some practitioners use normalisation with education to help patients recognise symptoms that can be associated with a heart attack, such as breathlessness, are also associated with physical activity;

“It shows how important it is to … try and help them not think that every symptom that they get then is going to relate to a heart attack, try to tell them that being out of breath is actually quite normal, when you have just done your stairs.” (4C—Exercise Facilitator).

#### Relaxation techniques are taught

All practitioners reported teaching relaxation techniques such as progressive muscle relaxation and guided imagery. Typically, practitioners *‘demonstrate the breathing exercises’* and *‘get the patients to practice it’* whilst in CR to ensure patients are using the correct technique. One practitioner described the prescriptive nature they teach relaxation as they inform patients, they

“need to practice it [relaxation], twice a day for three months to get the benefits from it.” (1B—Physiotherapist).

Relaxation is viewed positively by most practitioners;

“I think the relaxation ones [sessions] are very good” (4C—Exercise Facilitator),

with one practitioner linking their positive view of relaxation to the belief that supporting patients to reduce anxiety through relaxation may positively impact their ability to retain and apply the advice given.

‘If somebody is anxious they are not going to breath as deeply … they are not going to be listening when somebody is coming to discuss things with them … if they are sat there … all tense, you can then try to say by doing that you are not breathing as deeply so the oxygen is not getting to your heart … so we can teach some relaxation techniques … that can help you.” (5A—Nurse)

#### Barriers to patients’ psychological needs being met

All practitioners outlined barriers at different levels. At the practitioner level, low confidence coincided with organisational barriers such as not receiving any training to support patients’ psychological needs. There was the sense that practitioners recognise there is a need to provide psychological support but view what they can offer as

‘very simple … and not really as good as it should be.’ (2C—Nurse).

Further, practitioners reflected that they were limited in what they could offer due to limited time and other competing demands of the role.

“We don’t have a remit on how many patients we have coming in … so when you have got twenty patients you are obviously going to dilute what you do [psychologically] because you are mindful that you’ve got to see those twenty patients before you go home” (5A—Nurse).

At the service and commissioning levels, some practitioners have highlighted that CR is set up to prioritise patients’ physical recovery, and once patients are physically well, they are discharged even when psychological needs have not been met.

‘From a heart point of view they [the patients] are fine’ and “sometimes we [the practitioners] have discharged patients knowing physically they are fine but psychologically they are not.” (4C—Exercise Facilitator).

## Discussion

Practitioners recognise patients experience a range of cardiac and non-cardiac related worries and identify there is a need for psychological support with CR. Some practitioners expressed a lack of confidence and skills to discuss and support patients’ psychological needs, whilst also acknowledging a range of barriers including a lack of training, limited time, and service constraints. In turn, it could be inferred that patients’ psychological needs are not being met in a standardized approach across CR.

The insights from Study One was limited due to the relatively small sample of practitioners interviewed from CR services in the North-West of England. To enhance credibility and transferability, it is important to explore if the findings from Study One are understandable and relatable to a wider range of practitioners working across different CR services across England.

## Study Two

### Design

We employed the technique of member checking (31) for synthesised analysed data (30, 41) to assess the credibility (32) and transferability of the subthemes from Study One across a different sample of CR practitioners (32). This study considered the design used by Harvey (30), where participants were asked to provide feedback and add further comments on the overarching themes.

The epistemological stance underpinning the methodology was objectivism, which enabled the validation of results. As Study One was based on a small sample size in the Northwest of England, the initial findings cannot claim to be generalisable. Further qualitative exploration of the findings allows researchers to explore, clarify, and enhance the initial findings. To support this, participants also had the opportunity to reflect on their personal experiences and add any additional or different views. We believe that shared meanings can be developed through this qualitative research.

A qualitative research design with one-to-one semi-structured interviews was used. Study Two was a qualitative study nested within the NIHR-funded PATHWAY-Beacons Trial. PATHWAY-Beacons evaluates the implementation of group-MCT in CR services across the UK. Six CR services in England were enrolled in the implementation of the group-MCT.

### Participants

Purposive sampling was used to recruit participants because only practitioners enrolled in the PATHWAY-Beacons trial were eligible for inclusion. Recruitment was conducted via email, where potential participants were provided with an information sheet that outlined the study purpose. A total of 11 CR practitioners (six nurses, two physiotherapists, two exercise physiologists, and one health psychologist) were interviewed prior to training in the group-MCT. Most participants were female (91%), with an average age of 45.6 years (range, 27 years–61 years), and the average length of time working in CR was 11.6 years (range, 6 months to 24 years). Most practitioners had no prior training or experience in using group-MCT (82%). No participant withdrew from this study. The characteristics of practitioners are listed in **Table 3**.

**Table 3:** Study two participants characteristics.

Participant Number	NHS Site	Profession	Sex	Age	Length of time working in cardiology/CR (years)	Any previous mental health training (not MCT)	Prior training in group-MCT
1	A	Physiotherapist	M	49	10	Y	N
2	B	Nurse	F	57	15	Y	Y
3	C	Nurse	F	–	5	Y	N
4	B	Nurse	F	32	4	N	N
5	B	Nurse Consultant	F	56	24	N	Y
6	C	Exercise Physiologist	F	29	6	N	N
7	A	Nurse	F	61	18	Y	N
8	D	Nurse	F	47	18	N	N
9	E	Exercise Physiologist	F	27	5	Y	N
10	F	Physiotherapist	F	53	22	Y	N
11	E	Health Psychologist	F	–	0.5	Y	N

### Data collection

Three researchers (LW, AB, and LC) interviewed practitioners of MS Teams between August and October 2022 in the privacy of a work or home office. The verbal consent was recorded using an encrypted audio recorder. Interviews were audio-recorded and transcribed verbatim, with identifiable information removed, by a third-party organisation. On average, the interviews lasted 53 min, ranging from 30 min to 78 min, generating a total of 573.57 min of data.

A semi-structured interview guide was used. Initially, practitioners were asked to share their perspectives and experiences of the psychological support offered within the CR. Then, each practitioner was provided with a detailed verbal overview of the themes from Study One, whilst showing the themes, and subthemes visually over MS Teams. Practitioners were asked if the themes were understandable, resonated with their experiences, or were misrepresentative. Practitioners were also encouraged to add any additional views or experiences to explore their general agreement or disagreement with themes. The format of this question was inspired by that of Harvey (30). Further questions explored practitioners’ thoughts on the content of patients’ worries, their views on how men and women discuss mental health, and their confidence in supporting psychological difficulties. We continued the interview until thematic saturation, where saturation focused on the identification of new codes or themes (42).

### Ethical considerations

Ethical approval was granted by the UK Health Research Authority, Northwest Centre of Research Ethics Committee [REC reference: 22/HRA/2220]. All participants were provided with information on the study and provided verbal consent. We carefully considered how member checking was utilised throughout the research process.

### Data analysis

LW led the analysis and a constant comparative analysis was used to analyse the transcripts. LW systematically coded the transcripts, initially using the codes developed from Study One. The analysis in Study One generated 66 individual codes. Both deductive and inductive analyses were utilised as, when coding, 24 additional codes were added, which provided a deeper understanding of the themes, resulting in one theme being amended. Data were analysed to allow for further insights into the themes and subthemes to be explored and reported upon and developed in an iterative manner. The commonalities and contrasts in practitioner accounts across Studies One and Two were noted and discussed.

### Trustworthiness and reflexivity statement

To enhance trustworthiness, steps were taken to evidence the reflexivity, credibility, transferability, confirmability, and dependability of the study (31, 43, 44), as summarised in **Table 4**, which was adapted from Othman et al. (45).

**Table 4:** Strategies employed to enhance the trustworthiness of Study Two.

Trustworthiness Principles	Strategies	Examples across Study Two
Reflexivity	Critical reflection	• LW used a reflective diary throughout development, interviews, analysis and write up to enhance awareness of personal experiences and assumptions.
Dependability	Audit Trail	• All steps and procedures are described in the write up. • LW documented decisions made throughout the research process.
Confirmability	Diary	• LW’s reflective diary allowed for preconceptions, positioning, and assumptions to be noted throughout the research process and notes were made after the interviews.
Credibility	Prolonged engagement	• Interview guides were developed and refined by the research team to ensure questions were designed to assess the trustworthiness through synthesised data member checking. • Before the interviews, LC trained LW on using the interview guide, through a role play and provided feedback. • LW listened and re-listened to audio-recordings of the transcripts, as well as engaging in repeated reading of the transcripts.
	Iterative questioning	• Probes were asked to gather a more detailed understanding of participants responses with greater precision.
	Triangulation	• A range of different CR practitioners (physiotherapists, OT’s, exercise physiologist, nurses, psychologists) with various levels of experience were interviewed. These practitioners worked across different CR services in England. • Investigator triangulation—analysis decisions made by the research team.
Transferability	In depth description	• The sample selection and composition of the research team was outlined. • The researchers aimed to write thick analysis descriptions to facilitate transferability, as detailed findings allow readers to judge how applicable the findings to their own CR practice (Lincoln & Guba, 1985; Polit & Beck, 2014).
	Member checking of synthesised analysed data	• The interviews conducted in Study Two included a wider range of CR practitioners as LoBiono-Wood and Haber (2006) outlined, when the member check procedure is utilised on a different sample then findings can be used to assess transferability.

## Findings

**Figure 2:**
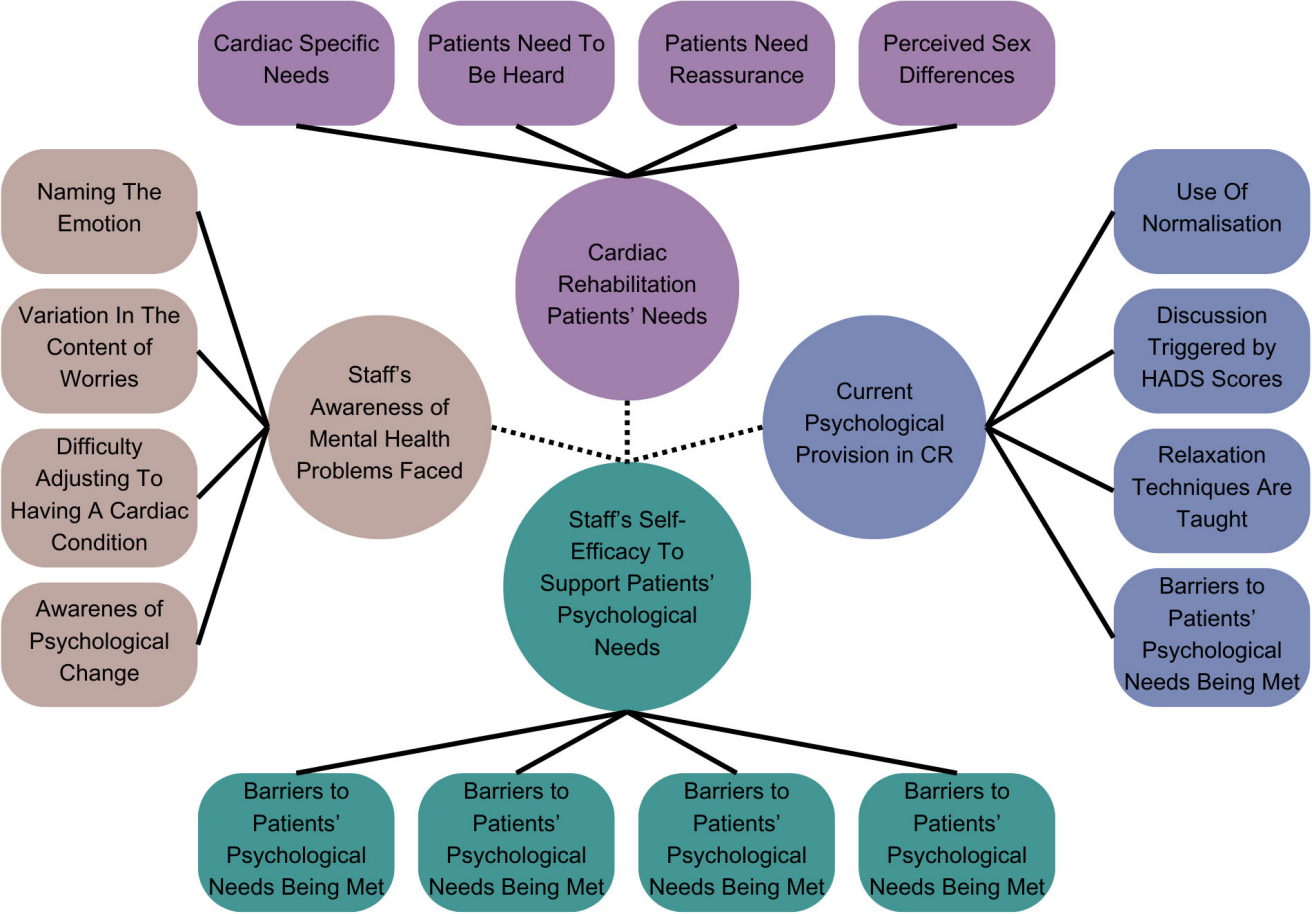
Visual overview of transferability across subthemes. Green: When coding, the codes for this subtheme were present in the transcript or practitioners expressed that they agreed with the subtheme. Orange: When coding, practitioners expressed that they somewhat agreed with the subtheme. Red: When coding, practitioners expressed that they did not agree with the subtheme. Grey: Codes for this subtheme were not present. *Not applicable as there were service changes due to the impact of the COVID-19 pandemic, which meant that the relaxation talk was not running.

Overall, the four overarching themes from Study One were found to be meaningful and relatable to a wider range of practitioners working in CR. **Figure 2** provides a visual overview of the findings, with each practitioner and subtheme listed. Green was assigned when practitioners explicitly agreed with the subtheme or if pre-existing codes from Study One were present. If practitioners found the subtheme somewhat representative, it was coded orange; if they disagreed with the subtheme, it was coded red. In instances where there was inconsistency across the subthemes; further insights were provided.

### Transferability (consistency) across subthemes

Seven subthemes were found to be consistent across all practitioners and are coded green in **Figure 2**, indicating high levels of transferability and credibility. The four subthemes also had high consistency across practitioners, with only one practitioner within each subtheme, expressing a slight variation in their experience. Transferability could not be consistently assessed for the subtheme *‘relaxation techniques are taught’* because five practitioners across two NHS sites did not deliver the relaxation talk following service changes in the pandemic. In relation to *‘awareness of psychological change*,*’* three practitioners did not comment on this subtheme; it was inferred that these practitioners may have felt unsure about this subtheme. **Table 5** provides an overview of the support quotes from Study Two. **Appendix 2** provides a summary of the number of practitioners in study two that confirmed the subthemes generated in Study One.

**Table 5:** Study two quotes evidencing consistency across subthemes.

Theme	Sub-Theme	Quotation
Staff’s awareness of mental health problems faced	Naming the emotion	• “I’d say about 98% of patients that sit in front of me have some kind of anxiety, confidence knocks, so there’s completely a huge spectrum of it.” (1A—Physiotherapist)
	Variation in the content of worries	• “We’ve got ranges of people worrying about dying to worried about going back to work, to people who are worried about paying the bills you know like it’s a big variety.” (4B—Nurse) • “The amount of people that talk about the difficulties in trying to get the tablets or getting the wrong tablets seems to be a massive problem but also it causes an awful lot of anxiety.” (8D—Nurse)
	Awareness of psychological change	• “They may say I’ve always been an anxious person … then they have a cardiac event which can magnify them you know what’s going on for them.” (7A—Nurse) • “A new diagnosis something cardiac and they develop all sorts of anxieties and a lot of them do get depressed.” (8D—Nurse)
	Difficulty adjusting to having a cardiac condition	• “I definitely agree with the difficulty adjusting to having a cardiac condition, I think that’s more so with the heart failure patients or not so much the defibrillators if they have an ICD or a pacemaker, they tend to be okay. I think they have quite a lot of support when they go into have the ICD fitted.” (1A—Physiotherapist) • “Absolutely, difficulty adjusting that’s huge, yes they do struggle with that.” (8D—Nurse)
Cardiac Rehabilitation patients’ needs	Cardiac specific needs	• “I think the needs of patients are quite widespread really … I think the patients with chronic health conditions need an acknowledgement of their worries and their concerns … they need an understanding of compliance to medication but also an understanding and recognising what their limitations are … and helping them come to terms with that … [and patients who have PCI] they just feel great afterwards and a few weeks after they’re back … smoking … [there’s a need] to help them to understand that lifestyle changes following these events will help to reduce further events in the future and I think it’s a skill trying to balance that without making people feel unnecessarily worried and focused on their health all the time.” (5B—Nurse Consultant).
	Perceived sex differences	• “I do think men struggle a bit more with it all because they’re macho and they’re you know, they can’t have mental health issues, they have to be strong … but I still do get the odd man who will admit to it.” (2B—Nurse)
	Patients need reassurance	• “We spend a lot more time talking to them trying to reassure them and do the best that we can.” (2B-Nurse) • “I think it’s spot on to be honest, especially with the reassurance needed … a lot of the time it’s just knowing that they can go back to doing the exercise that they were doing before and they’re going to be fine and that’s all the reassurance that they need.” (6C—Exercise Physiologist)
	Patients need to be heard	• “I think all they want to do is somebody to listen to them … if I sit and speak to somebody for half an hour well you can see the difference … because somebody has given them some time.” (8D—Nurse) • “I think needing to be heard is massive for the patient … because if you’re [the patient] sat with this feeling that you’re not being listened to, how are you going to trust that this team has my best interests at heart because you don’t actually know what I’m feeling or what’s going on.” (9E—Exercise Physiologist)
Staff’s self-efficacy to support patients’ psychological needs	Varying confidence levels in dealing with psychological issues	• “I definitely feel like we’ve got varying confidence throughout the team, my colleague … is a counsellor currently so she has that bit more confidence whereas some of the other nurses … always [have] that dread of having to do it [conversations around mental health] because of not knowing what comes up … that’s a major kind of variant throughout the team.” (1A—Physiotherapist)
	Uncertainty about the stress talk	• “I think in actually dealing with the problem it [the stress talk] did very little.” (4B—Nurse)
	Staff’s worries about negatively impacting on patients’ psychological wellbeing	• “Some staff more than others would think am I doing this right or are we making it worse and I think that would be highlighted more if a patient did get quite visibly upset you know in one of the talks, almost like oh have we opened a can of worms and we’re not qualified and trained to do that.” (7A—Nurse) • “I think this is always the worrying thing…, that you hit a nerve with somebody and what do you do when you open up that Pandora’s Box, isn’t it? … yes, I suppose that’s always a little bit of a worry.” (10F—Physiotherapist)
	Awareness of own professional boundaries	• “If I felt like I was you know getting out of my depth with something or something didn’t feel right then I would definitely refer on and I would definitely get support for something if I needed to, definitely.” (1A—Physiotherapist)
Current psychological provision in CR	Use of normalisation	• “If we were talking about [medication] side effects … saying it’s normal to feel like this, these are very common side effects and or if patients have open heart surgery talking about the healing process with them and again, that it’s normal … and that they know [anxiety is] it’s normal feeling what they’re feeling.’ (1A—Physiotherapist)
	Discussion triggered by the HADS scores	• “So, sometimes it’s done [the discussion] retrospectively … they fill it out, give it to us, we look at it like you know when they’re gone and then think oh no, their HADS are high I’ll have to give them a ring or something and just make sure everything’s okay or bring them back in again.” (6C—Exercise Physiologist)
	Relaxation techniques are taught	• “We’ve got our sort of box of relaxation techniques that we try and help people with, I think that’s one of our main resources.” (6C—Exercise Physiologist)
	Barriers to patients’ psychological needs being met	• “Like [limited] trained staff, time, patients’ willingness to open up, [lack of] resources.” (4B—Nurse) • “Lack of access to services, [not] having somebody trained in mental health within the team.” (5B—Nurse Consultant)

### Deeper insights

Interviews provided a deeper insight into the ‘*barriers to patients’ psychological needs being met.’* As previously identified, practitioners expressed knowledge and confidence deficits stemming from their limited training. Practitioners expanded on these barriers and reflected upon their impact on patients.

“I think you can’t truly get everything out of a patient … in terms of exercise and commitment if they’re not there psychologically … we feel like it’s almost one of those things that needs to be … prioritised before we go into the exercise … because they need to make sure they’re in a good place mentally.” (6C—Exercise Physiologist)

Recruiting practitioners across England highlighted the disparity in psychological provision across CR; some practitioners expressed no psychological support, whereas others had direct access to a psychologist. Further barriers include limited funding, lack of understanding of patients’ psychological needs from management, and service structure.

“I think one of the big ones is … higher management as well who’d help deliver funding is the lack of understanding and education on their level and the importance of it. Because I think even cardiac rehabilitation itself is poorly understood … so people then won’t understand the psychological aspects of it, especially if they’re at the decision-making level.” (7A—Nurse)

### Variable transferability (inconsistency) across subthemes

Three subthemes were deemed inconsistent across practitioners which included, ‘*perceived sex differences,’ ‘uncertainty about the stress talk,’ and ‘staff’s worries about negatively impacting on patients’ psychological wellbeing.’* Practitioners reported varying opinions across these themes.

#### Perceived sex differences

Some practitioners agree that there are sex differences and describe men as more hesitant to discuss their mental health. Many practitioners felt that this subtheme did not align with their clinical experiences, as they indicated that men were also open to talking about their mental health.

“My experience anyway is that men and women are equally happy to talk about what’s worrying them.” (3C—Nurse)

Despite these polarised views, all practitioners indicated that they observed individual differences, such as differences in cardiac conditions as well as sex, age, and personality differences. To capture the variation across a range of patients, the subtheme was modified and renamed *‘perceived individual differences.’*

“I’d say from my experience if people are anxious then they will say regardless of the sex, I think it’s more to do with personality than sex.” (5B—Nurse Consultant).

‘The younger patient, they’re more likely to be highly anxious in my experience … maybe the shock of it you know especially as you’re not expecting it as much as when someone’s quite elderly or they’re more likely to have younger children, you know a mortgage and all the rest of it.’ (7A—Nurse).

#### Uncertainty about the stress talk

There were inconsistencies regarding the value of delivering stress talks in CR. Some practitioners were unsure if the stress talk was effective at addressing patients’ needs;

“the talks are very just generic … some people have got a lot more psychological issues” (6C—Exercise Physiologist).

Whereas other practitioners viewed the stress talk more favourably and felt confident delivering it, particularly if they have been directly involved in writing the talk, routinely deliver it or received positive patient feedback;

“I think the more I do it the more confident I’ve felt with it [delivering the stress talk]’ (3C—Nurse).

Notably, it was evident that stress talk was not standardised across services, which may explain the varying views of practitioners. Some services have been required to reduce the length of their stress talk due to *‘finance’* and *‘capacity’* whereas others had recently re-developed their talks following funding for a psychologist to work in CR for 18 months.

#### Staff’s worries about negatively impacting on patients’ psychological wellbeing

Across practitioners, the majority expressed concern about negatively affecting patients’ well-being. However, this concern was not shared amongst all practitioners, particularly if they felt supported by a well-resourced multidisciplinary team.

“I feel like we would all sit down, we would do what we can, …so I don’t think we go away and think oh I hope I haven’t said that wrong or anything.” (1A—Physiotherapist).

One practitioner described how a lack of training and patients’ responses can impact practitioners’ concerns and confidence when discussing and trying to support psychological well-being.

“Some staff more than others would think am I doing this right or are we making it worse and I think that would be highlighted more if a patient did get quite visibly upset … like oh have we opened a can of worms and we’re not qualified and trained to do that’ (7A—Nurse).

## Discussion

The main themes and 11 subthemes from Study One, were transferable to a wider range of CR practitioners. Indicating most of the subthemes might be relevant to CR practitioners, however there was some variability within three subthemes; *‘uncertainty about the stress talk,’ ‘staff’s worries about negatively impacting on patients’ psychological wellbeing’ and ‘perceived sex differences,’* which was modified to *‘perceived individual differences.’* It could be inferred that these subthemes were less transferable; however, the variability was underpinned by a lack of training, funding, and capacity. As such, these subthemes may still be transferable to practitioners working in underresourced CR services. Notably, practitioners felt it could be beneficial for commissioners to develop their awareness of patients’ psychological needs to inform funding decisions and improve psychological support for patients.

## Overall discussion

This is the first study to explore CR practitioners’ understanding of CR patients’ psychological needs, practitioners’ confidence in supporting psychological needs, and their views on whether CR meets patients’ psychological needs. The present study was divided into Study One and Study Two. The four themes and associated subthemes generated from Study One, were based on a small sample, limiting transferability. Study Two aimed to address this limitation and found 11 subthemes to be transferable to a wider range of practitioners across England. Although the findings suggest transferability, it is important to recognise three subthemes received less consistent support; *‘perceived sex differences,’ ‘uncertainty about the stress talk,’* and ‘*staff’s worries about negatively impacting on patients’ psychological wellbeing.’* This indicates that practitioners across different CR services have varying views on these three subthemes.

Practitioners have described a range of psychological difficulties CR patients experience, including low mood, anxiety, and adjustment difficulties. Practitioners also noted that patients experienced a range of cardiac and noncardiac worries. This is *consistent with previous research, where CR patients noted that they experienced a range of worries* (27, 28, 46–48)*. However, this is the first time this have noted this CR phenomenon.* Practitioners noted that they aimed to ease patients’ concerns through listening and reassurance. Previous research evaluating cardiac patients’ needs noted that patients expressed a need to feel heard by practitioners (49).

Practitioners noted that some patients had an increased awareness of their bodily sensations associated with misinterpreting physical responses to exercise and/or anxiety as a sign of another heart attack, consistent with previous research where therapists noted misinterpretation of bodily sensations (50). These findings can be viewed from the perspective of the Metacognitive Model (51–54). In this model, monitoring for signs of another heart attack, worry, and seeking reassurance from practitioners are viewed as unhelpful coping strategies that are part of Cognitive Attentional Syndrome (CAS). Based on the metacognitive model, MCT focuses on regulating excessive and prolonged negative thinking and reducing CAS strategies that maintain anxiety, which reduces anxiety and depression in the CR population (35).

Although cognitive behavioural therapy (CBT) is recommended to treat anxiety and depression in cardiac patients (11, 55, 56), therapists are required to distinguish between realistic and unrealistic thoughts. This poses a challenge when the content of patients’ thoughts is associated with the consequences and potential risks of life-threatening cardiac events. However, MCT overcomes this therapeutic challenge, as it does not require the therapist to distinguish between realistic and unrealistic thoughts (57).

An additional challenge that needs to be considered when trying to integrate psychological care into CR is practitioners’ confidence levels. Our findings indicated that practitioners lack confidence in supporting patients’ psychological needs. They worried about negatively impacting their well-being, especially when patients were visibly upset, as they did not want to make patients feel worse. Practitioners felt apprehensive about asking patients about the content of their worries, as they wondered if engaging in their thoughts would maintain negative emotions. Practitioners also expressed apprehension about the stress talk and the effectiveness of relaxation techniques, as they felt they did not address patients’ most prominent concerns, anxiety, and worry. These findings align with those of a previous qualitative study in which CR patients did not view stress and relaxation techniques as beneficial (28).

Despite practitioners’ apprehension, they feel best placed to provide psychological support but require further training, resources, and support from their organisation, such as increased staffing, incorporating psychological support into job plans, and increasing funding. This is in line with BACPR standards (2023), suggesting that practitioners need to expand their roles to provide psychological support alongside their current clinical roles. Currently, there is disparity in the psychological support offered across CR services. Most offer stress and relaxation talks, whereas others may offer individual CBT (58). Owing to the modest effects of CBT on cardiac patients, other options should be sought. Recent evidence supports the use of metacognitive therapy alongside CR, which has been found to be associated with significant improvements in anxiety and depression compared with usual care (35).

For psychological care to be integrated into CR, tailored training for practitioners seems to be a priority to develop their skills and confidence. However, this should not be at the expense of specialist practitioners who are trained in evidence-based psychological interventions. This is of particular importance because, at present, there is no standardised psychological intervention on offer to address a range of worries and psychological symptoms, which our findings, along with other studies, have been found to be a prominent concern for patients.

### Strengths and limitations

This qualitative study was the first to document CR practitioners’ understanding of patients’ psychological needs. Only practitioners who consented to training in a group psychological intervention were interviewed. Practitioner positioning may have influenced their understanding and experiences, as some practitioners stated that they had a special interest in mental health and were motivated to improve service provision. Despite this, interviewing practitioners across England allowed us to explore the views of practitioners with real-world experience of working in different CR services and to evaluate the consistency of practitioners’ views. While the findings from Study Two, highlight most subthemes were transferable, it is important to acknowledge that practitioners’ responses may have been influenced by their level of engagement with the synthesised data from Study One. As such, steps were taken to facilitate their engagement, such as using accessible language and a visual image of the themes, utilising open questions, and encouraging practitioners to express confirming or disconfirming views.

### Implications for policy and practice

Specific recommendations for practice include ensuring that all CR practitioners have the skills and confidence to recognise patients with varying psychological needs. This can be facilitated through active listening, compassionate communication, and confidence in emotive conversations. To support this, all practitioners would benefit from training on how to have conversations on mental health. Within CR, it would be beneficial to have several practitioners who engage in further training in evidence-based interventions, such as MCT, to enhance their capabilities to deliver psychological interventions to support the diverse worries and symptoms experienced by patients. Psychologists are a limited resource in CR; therefore, it is paramount that psychologists utilise their skills in indirect work to provide tailored training, consultation, and supervision to the wider CR team. Moving forward, it could be beneficial for future commissioning decisions to consider patients’ psychological needs as well as the barriers practitioners face, to enable CR to better support patients, with the goal of improving patient outcomes.

## Conclusion

The present study offers an exploration of CR practitioners’ understanding of CR patients’ psychological needs, their concerns when faced with psychological distress, and their experiences of the current psychological provision offered in CR. Utilising two qualitative samples allowed for a wider exploration in an under-researched area. Overall, most themes and subthemes were credible and transferable. The findings provide valuable insights into practitioners’ understanding and indicate training needs as well as wider organisational barriers that could be addressed. The current findings suggest that CR patients experience a range of worries and psychological difficulties, which can be viewed from the perspective of the metacognitive model. The findings can be used to initiate discussions about how CR services and CR practitioners, including clinical psychologists, may develop roles and provisions to best meet the psychological needs of CR patients.

## Data Availability

The raw data supporting the conclusions of this article is available upon request to the corresponding author.
