# Supplementary material for: Cardiac Rehabilitation practitioners’ views on patients’ psychological needs: a qualitative study
Source: Front Psychiatry. 2024 Oct 3;15:1434779. doi: 10.3389/fpsyt.2024.1434779 (PMC11484254; doi:10.3389/fpsyt.2024.1434779)
Supplement: Supplementary file 2 [file Table2.docx]

**Appendix 2: Graph to show number of CR Practitioners from Study Two who confirm the sub-themes generated in Study One.**
